# Potential Foraging Decisions by a Desert Ungulate to Balance Water and Nutrient Intake in a Water-Stressed Environment

**DOI:** 10.1371/journal.pone.0148795

**Published:** 2016-02-19

**Authors:** Jay V. Gedir, James W. Cain, Paul R. Krausman, Jamison D. Allen, Glenn C. Duff, John R. Morgart

**Affiliations:** 1 Department of Fish, Wildlife and Conservation Ecology, New Mexico State University, Las Cruces, New Mexico, United States of America; 2 U.S. Geological Survey New Mexico Cooperative Fish and Wildlife Research Unit, Department of Fish, Wildlife and Conservation Ecology, New Mexico State University, Las Cruces, New Mexico, United States of America; 3 School of Natural Resources and the Environment, University of Arizona, Tucson, Arizona, United States of America; 4 Department of Animal Sciences, University of Arizona, Tucson, Arizona, United States of America; 5 United States Fish and Wildlife Service, Cabeza Prieta National Wildlife Refuge, Ajo, Arizona, United States of America; Universidade de Aveiro, PORTUGAL

## Abstract

Arid climates have unpredictable precipitation patterns, and wildlife managers often provide supplemental water to help desert ungulates endure the hottest, driest periods. When surface water is unavailable, the only source of water for ungulates comes from the forage they consume, and they must make resourceful foraging decisions to meet their requirements. We compared two desert bighorn sheep (*Ovis canadensis nelsoni*) populations in Arizona, USA: a treatment population with supplemental water removed during treatment, and a control population. We examined whether sheep altered their seasonal diets without supplemental water. We calculated water and nutrient intake and metabolic water production from dry matter intake and forage moisture and nitrogen content, to determine whether sheep could meet their seasonal daily water and nutrient requirements solely from forage. Diets of sheep were higher in protein (all seasons) and moisture (autumn and winter) during treatment compared to pretreatment. During treatment, sheep diet composition was similar between the treatment and control populations, which suggests, under the climatic conditions of this study, water removal did not influence sheep diets. We estimated that under drought conditions, without any surface water available (although small ephemeral potholes would contain water after rains), female and male sheep would be unable to meet their daily water requirements in all seasons, except winter, when reproductive females had a nitrogen deficit. We determined that sheep could achieve water and nutrient balances in all seasons by shifting their total diet proportions by 8–55% from lower to higher moisture and nitrogen forage species. We elucidate how seasonal forage quality and foraging decisions by desert ungulates allow them to cope with their xeric and uncertain environment, and suggest that, with the forage conditions observed in our study area during this study period, providing supplemental water during water-stressed periods may not be necessary for desert bighorn sheep.

## Introduction

The four basic requirements for animal survival are food, water, cover, and space, with food and water being of more immediate necessity. Environmental context largely dictates forage conditions and water availability, which can vary widely. Animals must make practical foraging decisions to enable them to meet their daily food and water requirements under the specific resource constraints of their environment. For example, in a nutrient-deficient environment, ungulates are restricted by their gut capacity and rumination time [1], and thus must select forages with a nutritional quality that allows them to meet their nutritional requirements given these limitations. Alternatively, in an arid environment, when free-standing water is scarce, individuals may be forced to select forages with high moisture content to meet their necessary water intake [2]. Often, forages with the highest moisture content, are also lower in protein (e.g., succulents) [3, 4], which potentially creates a trade-off where herbivores struggle to meet both water and nutrient requirements. Thus, resourceful forage selection is essential to species fitness.

For ungulates inhabiting arid environments, scarcity of water and variable precipitation patterns make water a particularly important influence on survival and reproduction, and ultimately, on population dynamics [5–8]. In addition to determining surface water availability, precipitation also influences forage quality and quantity. The unpredictability in the temporal and spatial distribution of rainfall in these arid regions, can force animals at times to rely solely on preformed water in their forage to meet their daily requirements.

Desert-dwelling ungulates have evolved physiological adaptations to enable them to cope in their xeric environment (e.g., lowering respiratory and metabolic rates, varying body temperature, producing low moisture feces and highly concentrated urine [9]). In addition, their herbivory can allow them to inhabit arid regions through behavioral adaptations like diet selection, which can be as effective as physiological adaptations [10]. Some species developed these adaptations to an extent whereby they can survive without drinking water for extended periods (e.g., Arabian oryx [*Oryx leucoryx*], [11]; Grant’s gazelle [*Gazella granti*], [12]; springbok [*Antidorcas marsupialus*], [13]; cape eland [*Taurotragus oryx*], [14]; dik-dik [*Rhynchotragus kirki*], [15]; fringe-eared oryx [*Oryx beisa callotis*], [14, 16]). For these species, their daily water requirements would be fulfilled solely from preformed water obtained from ingested forage and metabolic water production.

However, most species living in arid environments have at least some dependency on surface water to maintain water balance. For many of these species, as a contingency during periods of scarce water availability, a long-standing practice of wildlife managers has been to provide water through wildlife water developments [17–21]. These artificial water points can potentially serve to increase productivity and range carrying capacity, and manipulate or expand population distributions [22, 23]. It is costly and time-consuming to construct and maintain these water catchments [19], and few empirical data are available to assess the necessity or efficacy of water provision for many desert species.

The desert bighorn sheep (DBS, *Ovis canadensis nelsoni*) is a desert-adapted ungulate that can go for long periods without drinking, but controversy surrounds this species, as to whether they can survive year-round without surface water. Some have reported DBS surviving in areas without permanent water sources [24–28]. In contrast, others have documented that, at least in summer (the driest period of the year), DBS are mostly found within 3 km of drinking water [29–34]. Consequently, it is generally considered that water sources are one of the essential habitat components for nearly all DBS populations [34–38]. In response to extirpations and significant population declines last century [39, 40], and as part of a recovery strategy, wildlife managers have been providing water across DBS range via modified natural and manmade catchments for nearly 50 years. The debate continues as to whether water provision is necessary for or is beneficial to DBS populations.

To gain an understanding of DBS relationship with water availability, we examined the impact of cessation of water provision on their diet selection and nutritional quality. We assessed whether DBS can maintain water and nutrient balance (particularly during the hottest, driest periods and during drought) without free-standing water. We first examined whether the removal of supplemental surface water affected DBS diet selection. We then estimated DBS water and nitrogen (N) intake to test whether seasonal forage quality and quantity, and DBS physiological capacity allows them to acquire their daily water and nutrient requirements solely from forage. We examined whether foraging decisions by DBS could potentially enable them to survive without permanent water sources, by conducting a theoretical experiment where we test hypothetical diet shifts at a forage species level. We predicted that DBS could meet their seasonal daily water and nutrient requirements solely from the forage they consume. Findings from this study will further our understanding of how water availability and climatic conditions influence forage quality and foraging decisions by desert ungulates, and the impact of wildlife water developments on DBS.

## Materials and Methods

### Study species

Desert bighorn sheep inhabit arid lands in the southwestern United States and northern Mexico, and occur on rugged mountain ranges, which are often isolated by broad valleys or plains [41]. Diets of DBS are a diverse mix of forbs, shrubs, trees, grasses, and succulents, and have high temporal and seasonal variability [42, 43]. Desert bighorn sheep have a protracted breeding season, ranging from July to December [44]. Their gestation period is about six months and they typically give birth to a single lamb [45].

From 2002–2004, 37 adult female DBS were captured by Arizona Game and Fish Department personnel with a net gun fired from a helicopter [46] (chase time was limited to 10 minutes), fitted with global positioning system telemetry collars (900 g; models 440 and 3580, Telonics, Mesa, Arizona), and released at the point of capture within 15 minutes. Collars were programmed to record a location every 13 hours and locations were transmitted via the Argos satellite system every three days (Service Argos, Largo, Maryland). Subsequent DBS were collared to replace those lost to mortality and collars with expired batteries, such that 6–10 radio-collared DBS on each mountain range (see next section) were maintained throughout the study. All capture and handling procedures followed acceptable methods [47] and were approved by the University of Arizona Animal Care and Use Committee (Protocols 01–191 and 04–180).

### Study area and experimental design

The Cabeza Prieta National Wildlife Refuge (CPNWR) is 3,480 km^2^ in the Sonoran Desert in southwestern Arizona, USA, on the international border with Mexico. The area consists of a series of rugged mountain ranges from 200–900 m in elevation. Large alluvial fans (bajadas) surround the base of these mountains, and ranges are separated by wide alluvial valleys.

We compiled long-term (1970–2005) precipitation data from the weather station nearest the study area (Tacna, Arizona, approx. 64 km north; [48]). Based on long-term precipitation and temperature patterns, seasons were defined as winter (January to March), early summer (April to June), late summer (July to September), and autumn (October to December). Mean annual precipitation is 101 ± 9 mm, with high inter-annual variability (CV = 56%), and peaks in late summer and winter (mean seasonal precipitation ± SE: winter – 36 ± 5 mm; early summer – 6 ± 2 mm; late summer – 36 ± 6 mm; autumn – 24 ± 4 mm). In summer, mean daily low temperature is 22°C and mean daily high temperature is 41°C, but high temperatures commonly exceed 45°C in summer. In winter, mean daily low and high temperatures are 3°C and 21°C, respectively [48]. We defined precipitation periods using the Standardized Precipitation Index (SPI; [49, 50]), which is the number of standard deviations that observed cumulative precipitation deviates from the long-term average. We calculated the 3-month SPI for each month from 2002 to 2005 using program SPI SL 6 [51]. Seasons were classified as drought (when SPI was consistently negative and ≤ -1, beginning when SPI fell below 0, and ending when SPI became positive), average (SPI fluctuated near 0 and did not become consistently positive or negative), or above average (when SPI was consistently positive and ≥ 1).

Characteristic vegetation on the mountains is ironwood (*Olneya tesota*), catclaw acacia (*Senegalia greggii*), foothill palo verde (*Parkinsonia microphyllum*), white bursage (*Ambrosia dumosa*), ratany (*Krameria* spp.), brittlebush (*Encelia farinosa*), Wright’s buckwheat (*Eriogonum wrightii*), and mallow (*Sphaeralcea* spp. and *Hibiscus* spp.). Typical vegetation in the valleys is creosote bush (*Larrea tridentata*), white bursage, and ocotillo (*Fouquieria splendens*), with ironwood, blue palo verde (*P*. *florida*), and triangle-leafed bursage (*A*. *deltoidea*) common along washes. Prevalent grasses and forbs include three-awn (*Aristida* spp.), grama (*Bouteloua* spp.), big galleta (*Pleuraphis rigida*), globe mallow (*Sphaeralcea* spp.), indian wheat (*Plantago patagonica*), and lupine (*Lupinus* spp.). Common cacti include giant saguaro (*Cereus giganteus*), barrel cactus (*Ferocactus* spp.), fishhook cactus (*Mammillaria* spp.), teddy bear cholla (*Cylindropuntia bigelovii*), buckhorn cholla (*C*. *acanthocarpa*), and chain fruit cholla (*C*. *fulgida*).

The study sites were the Sierra Pinta (SP) and Cabeza Prieta (CP) mountains located in the western half of CPNWR. Precipitation results in ephemerally flowing desert washes and natural rock depressions (tinajas) that are temporarily filled with water. The SP has three water catchments and CP has four located in DBS habitat, and these provide the only known perennial sources of water. The area is described in detail in Cain et al. [52].

We used a before-after–control-impact design [53, 54] to examine the effects of water provision on DBS diet selection and quality. The SP was the treatment range and CP the control range. During the pretreatment period (February 2002 to February 2004), U.S. Fish and Wildlife Service and Arizona Game and Fish Department hauled water when necessary to ensure that all water catchments on both mountain ranges contained water year-round. From March 2004 to October 2005 (treatment period), all three water catchments on SP were drained and float switch-activated pump systems were installed to ensure they remained dry for the duration of the treatment period.

### Forage sample collection and analyses

Forage sampling locations were selected using GPS positions taken from peak morning and afternoon DBS foraging periods, adjusted for seasonal changes in foraging activity due to shifts in timing of daylight hours. These plots were used to assess DBS forage availability and collect representative forage samples for nutritional analyses. Only GPS locations within the previous two days for the appropriate time periods from 8–10 randomly selected DBS in each mountain range were used. Forage samples were collected from ten forage plots on each range during a 4–5 day period in the middle of each season (i.e., February, May, August, and November). A modified line-intercept method was used to estimate percent cover of each forage species [55, 56] using four 30-m transects originating from the center of each plot. The direction of the initial transect was based on a randomly generated compass bearing, and the remaining transects started at 90°, 180°, and 270° angles from the first transect. All vegetation intercepting each transect was measured and species percent cover estimated by dividing the total intercept length covered by each species along all four transects by the total length of all transects [55, 56]. A detailed description of forage sampling methods is available in Cain et al. [52].

In each season, ≥ 100 g of each of the following DBS forage species were collected to determine moisture and nutritional content: desert agave (*Agavi deserti*), barrel cactus, big galleta, brittlebush, catclaw acacia, desert lavender (*Hyptis emoryi*), fishhook cactus, globe mallow, ironwood, foothill palo verde, ratany, ephedra (*Ephedra aspera*), silverbush (*Ditaxis lanceolata*), three-awn, Wright’s buckwheat, and white bursage. These species constituted 73–98% (mean = 85.4%; SD = 7.9%) of the seasonal diets of DBS in SP and CP in 2002–2005 (J.W. Cain, unpublished data). All forage samples were composites of ≥ 4 individual plants unless there was < 4 individuals in the plot. Fresh weight of all forage samples were determined immediately upon collection.

Forage samples collected for nutritional analyses were dried at 50° C in a drying oven (Model 320, National Appliance Company, Portland, Oregon) to determine dry weight, and moisture content of each forage species estimated. Dried forage samples were ground through a Wiley mill with a 1 mm mesh screen. Nitrogen content was determined using a TC400-N Analyzer (Leco FP528, Leco Corp., St. Joseph, Missouri). Neutral detergent fiber (NDF) and acid detergent fiber (ADF) content were determined following the Van Soest method [1] using an Ankom^200^ Fiber Analyzer (Ankom Technology, Macedon, New York). Ash content was determined by placing dried samples in a muffle furnace at 500°C for 4 hours and weighing residual ash.

### Diet composition and selection

In each season, 10–20 female DBS pellet groups were collected from each mountain range to estimate diet composition using microhistological analysis. Twenty randomly selected microscope fields were sampled from each of three slides per pellet group [57–59]. Forage species were identified using characteristics of the epidermis and cuticle, and frequency, particle density, and percent composition of each species determined [57, 60]. It was assumed that potential biases from differential digestibility among forage species would equally affect estimates of diet composition for both mountain ranges and treatments [61–63].

Microhistological analyses identified 24 forage species consumed by DBS in our study area. In addition to the 16 forage species listed above, wolfberry (*Lycium* spp.), ocotillo (*Fouquieria splendens*), canyon ragweed (*Ambrosia ambrosoides*), lupine (*Lupinus* spp.), bladder sage (*Scutellaria mexicana*), bedstraw (*Galium stellatum*), janusia (*Janusia* spp.), and fairy duster (*Calliandra eriophylla*) were also present in DBS diets. These plant species represented > 80% of DBS diets in the study area (J.W. Cain, unpublished data). Forage plant types were forb (silverbush, globe mallow, bedstraw, janusia, and lupine), grass (big galleta and three-awn), shrub (brittlebush, white bursage, ephedra, desert lavender, ratany, Wright’s buckwheat, canyon ragweed, fairy duster, bladder sage, wolfberry, and ocotillo), succulent (barrel cactus, fishhook cactus, and desert agave), and tree (catclaw acacia, foothill palo verde, and ironwood).

Selection of forage types by DBS (see Diet composition and selection in Results) was evaluated using Jacobs’ modified electivity D index (Jacobs’ D; Eq 2; [64]). This is a modification of Ivlev’s electivity index E [65], and is less sensitive to sampling errors for rare species [66].

$$D_{i}=\frac{r_{i}-p_{i}}{r_{i}+p_{i}-2r_{i}p_{i}}$$ (1)

Here D_i_ is the Jacobs’ D index value for forage type i, r_i_ is the proportion of forage type i in DBS pellets (i.e., diet) and p_i_ is the proportion of forage type i in the environment (i.e., availability). Jacobs’ D values range from -1 to 1, where negative and positive values indicate species avoidance and preference, respectively.

### Water and nutrient balance

Seasonal DBS water and nutrient intake in relation to requirements were examined on SP during the treatment period (i.e., when water catchments were maintained empty). Only pellet groups in which identified forage species made up ≥ 90% of the total diet composition were used to calculate seasonal water and nutrient intake. For estimations of water and nutrient intake of the unknown portion of the diet, we used seasonal mean water and N content by plant type from the known portion of the diet, and at seasonal proportions found in the known portion of the diet. Seasonal water and nutritional content of forage species not analyzed and lipid content of all forage species were taken from the literature [4, 60, 67–72]. We determined water and nutrient balance for non-reproductive females, reproductive females, and males. The DBS lambing season lasts from late December to early April [73], with peaks in January and March [73–75]. Therefore, we designated reproductive females as early breeders (i.e., late gestation and early lactation correspond with autumn and winter, respectively) and late breeders (i.e., late gestation and early lactation correspond with winter and early summer, respectively).

We used seasonal dry matter intakes (DMI) from Mazaika et al. [76], where captive desert bighorn males were fed seasonal native browse, forbs, and grasses collected from local DBS range. Mean mass of 78 kg and 52 kg for desert bighorn males and females, respectively, were considered representative of southwestern Arizona, as reported in the nearby Kofa National Wildlife Refuge [74]. Therefore, daily DMIs for a 78 kg male were 3.87 kg in early summer, 2.66 kg in late summer, 2.91 kg in autumn, and 4.25 kg in winter [76]. Thus, after adjusting for metabolic weight, daily DMIs for a 52 kg non-reproductive female were 2.86 kg in early summer, 1.96 kg in late summer, 2.15 kg in autumn, and 3.14 kg in winter. A study on domestic sheep showed that voluntary DMI of reproductive females remained unchanged during late gestation and increased by 17% during lactation [77], and thus, we used DMIs of 3.67 kg/day for early breeding females in winter and 3.35 kg/day for late breeding females in early summer to account for increased forage intake during lactation.

Daily water intake in the absence of free-standing water is the total of preformed water (i.e., water contained in forage) and metabolic water produced. Daily preformed water intake was estimated using the following equation:

$$\mathrm{Preformed water intake}=\sum_{s}^{1}\left(\mathrm{W x D x DMI}\right)$$ (2)

where s is number of forage species in diet, W is moisture content of each forage species, and D is proportion of forage species in diet. Metabolic water produced was estimated from the following equation [78, 79]:

$$\mathrm{Total metabolic water}=\sum_{s}^{1}\left(0.40P+1.07L+0.56C\right)$$ (3)

where s is number of forage species in diet, P is crude protein (g; N x 6.25), L is lipids (g), and C is total carbohydrates (g), calculated from the following equation:

Total carbohydrates=1−(L+(NDF−ADF)+Ash+P) (4)

We assumed utilizable metabolic water by DBS to be about half of the total metabolic water produced, after accounting for water lost through excretion (urination and defecation), from respiration, and evaporation from the body surface [78].

Previous water deprivation trials determined that daily maintenance water requirements for DBS were 4% of bodyweight during early summer and 3% of bodyweight in the remaining seasons [80]. Research on Awassi (*Ovis aries*), a desert-adapted sheep breed, showed that daily water turnover rates in females increased by 11% and 30% during late gestation and lactation, respectively [81]; we adjusted minimum daily water requirements for reproductive females accordingly. Daily N requirements for DBS were 0.89% DMI for maintenance in all seasons for non-reproductive females and males, and 1.5% DMI during late gestation and lactation for reproductive females [82, 83].

### Statistical analyses

To test for the impacts of water removal on DBS diet selection and quality of forage consumed, we compared parameters among ranges (i.e., SP treatment and CP control) and treatment periods. We used generalized linear models to test for differences in diet composition (by plant type) and forage quality (moisture and N content) among ranges, seasons, and treatment periods. Diet data were logit-transformed prior to analyses to meet parametric assumptions of normality and homogeneity of variance [84]. Estimated marginal means and 95% confidence intervals of transformed data were back-transformed for presentation. We used SPSS 21.0 [85] for statistical analyses. Means are reported with standard errors for descriptive statistics and probabilities of α < 0.05 were accepted as significant.

## Results

### Forage and diet quality

In all seasons, grasses had the lowest mean moisture content (range: 18 ± 3.1–37 ± 3.0%), succulents had the highest (76 ± 1.5–82 ± 1.7%), and moisture levels were moderate in shrubs (37 ± 1.1–52 ± 1.1%), forbs (33 ± 2.6–52 ± 2.1%), and trees (43 ± 2.5–51 ± 2.2%). Grasses and succulents had the lowest mean N content in all seasons (grasses: 0.7 ± 0.13–1.0 ± 0.12%; succulents: 0.8 ± 0.07–0.9 ± 0.06%). Trees had the highest N content in early summer (1.9 ± 0.08%), whereas forbs contained the most N in winter (2.2 ± 0.09%).

**Fig 1:**
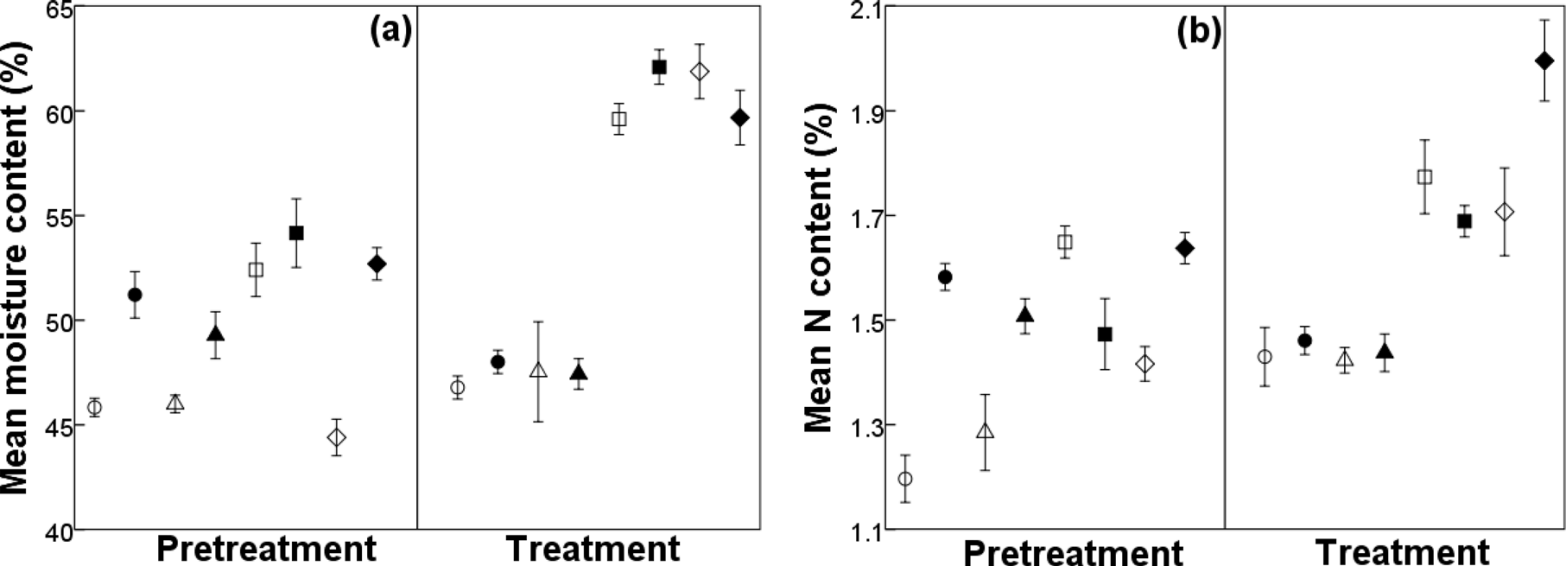
**Mean (± 95% confidence interval) seasonal a) moisture content and b) nitrogen (N) content of female desert bighorn sheep diet during pretreatment and treatment periods on Sierra Pinta (treatment; open symbols) and Cabeza Prieta (control; closed symbols), Cabeza Prieta National Wildlife Refuge, Arizona, USA, from 2002 to 2005.** Seasons are early summer (circles), late summer (triangles), autumn (squares), and winter (diamonds).

Mean moisture content of forage consumed by DBS differed between ranges (*F*_1, 410_ = 30.23, *P* < 0.001) and among seasons (*F*_3, 410_ = 91.06, *P* < 0.001). During the treatment period (i.e., when water was removed from catchments on SP), moisture content of DBS diets on SP was similar to pretreatment during summer, and was higher than during pretreatment in autumn (14%) and winter (39%; Fig 1A). On CP during treatment, DBS forage moisture levels were also higher than during pretreatment in autumn (13%) and winter (12%), but were lower than pretreatment in early (7%) and late (4%) summer (Fig 1A). Consequently, these changes resulted in the water content of DBS diets on SP and CP being similar during treatment in all seasons, with the exception of autumn where it was slightly higher on CP (Fig 1A). During pretreatment, water in DBS diets was lowest during winter and late summer on SP and CP, respectively; however, during treatment, it was lowest during summer (early and late) on both ranges (Fig 1A).

Mean N content of DBS diets differed by range (*F*_1, 410_ = 33.29, *P* < 0.001), season (*F*_3, 410_ = 67.35, *P* < 0.001), and season within range (*F*_3, 410_ = 25.56, *P* < 0.001). During pretreatment, N content of forage consumed by DBS on SP was lower than that on CP in summer (early and late) and winter, and higher during autumn (Fig 1B). Nitrogen content of DBS forage on SP was higher during treatment compared to pretreatment in all seasons (range 8–21%), whereas on CP, N levels were lower during treatment than during pretreatment in early (8%) and late (5%) summer, and higher in autumn (15%) and winter (22%; Fig 1B). Consequently, these changes resulted in N content of DBS diets during treatment being similar between ranges in all seasons except winter (17% higher on CP; Fig 1B). Within season, DBS diets on SP had greater N levels in autumn and winter than in summer during both the pretreatment and treatment periods (range 10–38%; Fig 1B); DBS on CP exhibited the same pattern during treatment (i.e., range 18–39% greater N content in autumn and winter). However, during pretreatment, N levels in DBS diets were greater in early summer and winter, than during late summer and autumn (range 5–11%; Fig 1B).

On both ranges during the treatment period, DBS diets were higher in water and N content in autumn and winter, than during summer (Fig 1A and 1B).

### Diet composition and selection

**Fig 2:**
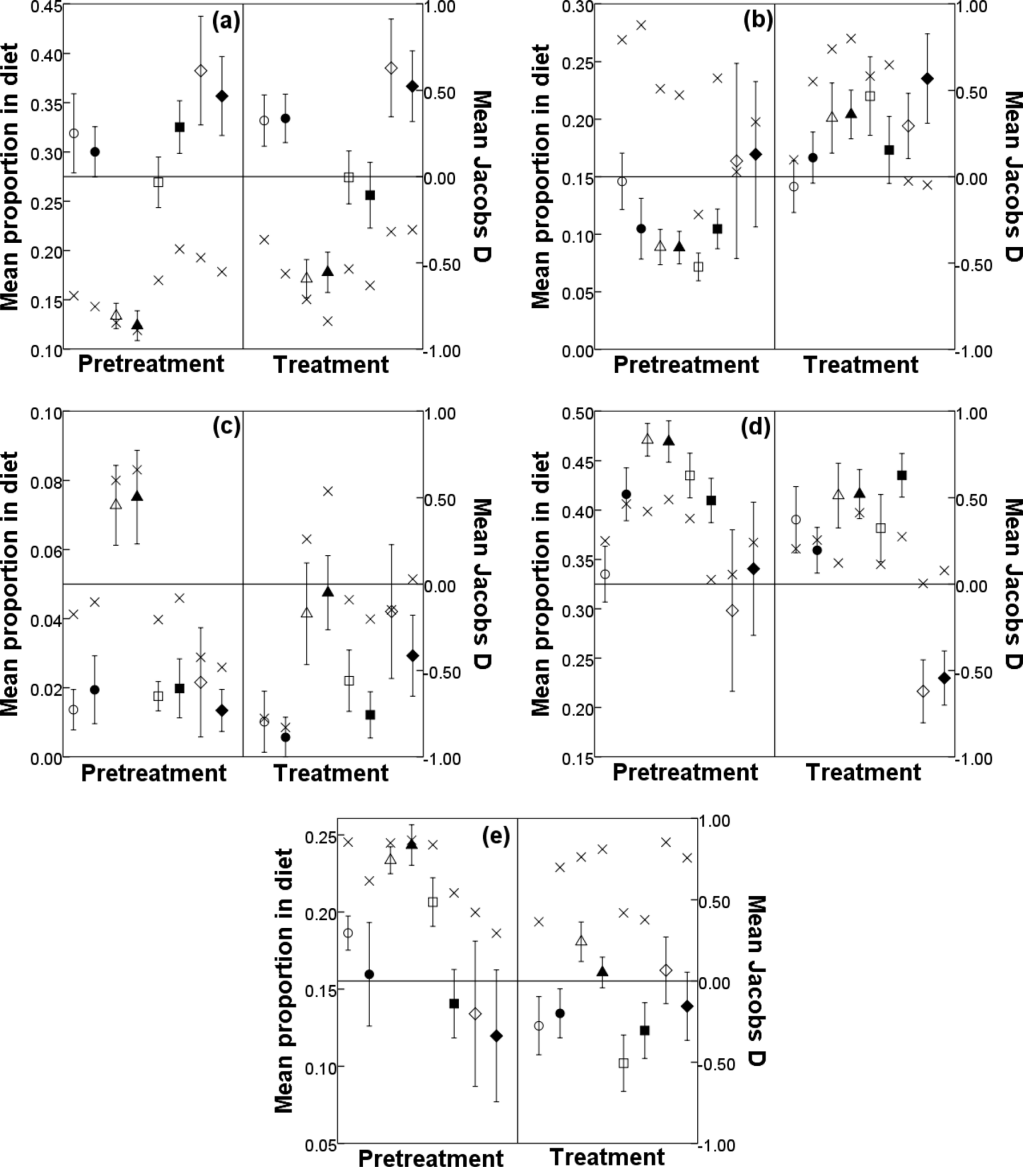
**Mean (± 95% confidence interval) seasonal proportion in female desert bighorn sheep diet of a) shrubs, b) forbs, c) grasses, d) trees, and e) succulents during pretreatment and treatment periods on Sierra Pinta (treatment; open symbols) and Cabeza Prieta (control; closed symbols), Cabeza Prieta National Wildlife Refuge, Arizona, USA, from 2002 to 2005.** Seasons are early summer (circles), late summer (triangles), autumn (squares), and winter (diamonds). Jacobs D index values (‘X’ symbols) indicate preference (> 0) and avoidance (< 0) of forage types.

The proportion of each forage type in DBS diets did not differ between ranges within seasons (F_3, 410_ = 0.21–1.84, P = 0.139–0.892). During pretreatment and treatment, the proportion of shrubs in DBS diets was lowest during late summer and highest during winter (Fig 2A). Shrub consumption by DBS demonstrated an increasing trend from late summer to autumn to winter during pretreatment and treatment on both ranges (Fig 2A). The proportion of forbs in DBS diets exhibited an increasing trend over the study period (i.e., from pretreatment through treatment); however, forb preference remained unchanged (Fig 2B). Grass has a very low moisture content, and although consistently making up a very small part of DBS diets, grass consumption on both ranges during treatment decreased by 46–56% in late summer (Fig 2C). Trees made up a relatively large portion of DBS diets across ranges, seasons, and treatments (30–47%), with the exception of winter during treatment, where the proportion of trees consumed by DBS fell below 25% (Fig 2D). Surprisingly, succulent consumption by DBS on SP decreased during the treatment period in all seasons (23–51%), except winter, whereas on CP, late summer was the only season in which the proportion of succulents in the diet of DBS decreased (34%; Fig 2E). Desert bighorn sheep exhibited preferences for forbs, trees, and succulents, and selected shrubs and grasses at proportions lower than availability in almost all seasons across ranges and during both pretreatment and treatment periods (Fig 2).

### Water and nutrient balance

We estimated DBS water and nutrient balances on SP (treatment range) during the treatment period, when water catchments were maintained empty. When modelling water and nutrient balances, we assumed there was no surface water available to DBS during all seasons. We estimated DBS daily nutrient and preformed water intake, and metabolic water production for each season based on diet composition, forage quality, and DMI. We assumed that DBS diets did not differ between sexes [86, 87], and thus, female diet compositions measured in this study were also used to calculate male intakes. Early and late summer were the only seasons where we predicted DBS (both sexes) would have a negative daily water balance (Table 1). In all seasons, sexes, and reproductive states (early and late breeders), there was a surplus of daily N intake, with the exception of early summer, when we estimated that late breeding females (during early lactation) would have a slightly negative N balance (Table 1).

**Table 1:** Estimated seasonal daily water and nitrogen (N) balance of desert bighorn sheep non-reproductive females, reproductive females (early and late breeders), and males on Sierra Pinta (treatment range) during the treatment period in Cabeza Prieta National Wildlife Refuge, Arizona, USA, from 2002 to 2005. Intakes are calculated from dry matter intakes reported in Mazaika et al. [76], and forage moisture and N content measured in this study.

		Water maintenance (ml)				N maintenance (g)			
		Female	Reproductive female		Male	Female	Reproductive female		Male
Season	Parameter		Early	Late			Early	Late	
Early summer	Intake–average precipitation^a^	1970	1970	2305	2666	41.4	41.4	48.4	56.0
	Requirement^b^	2080	2080	2704	3120	25.5	25.5	50.2	34.4
	Balance	-110	-110	-399	-454	15.9	15.9	-1.8	21.6
Late summer	Intake–drought^c^	1310	1310	1310	1778	29.4	29.4	29.4	39.9
	Requirement	1560	1560	1560	2340	17.4	17.4	17.4	23.7
	Balance	-250	-250	-250	-562	12.0	12.0	12.0	16.2
Autumn	Intake–high precipitation^d^	1760	1760	1760	2383	36.1	36.1	36.1	48.8
	Intake–drought^e^	1501	1501	1501	2031	36.4	36.4	36.4	49.3
	Requirement	1560	1732	1560	2340	19.1	32.3	19.1	25.9
	Balance–high precipitation^d^	200	28	200	43	17.0	3.8	17.0	22.9
	Balance–drought^e^	-59	-231	-59	-309	17.3	4.1	17.3	23.4
Winter	Intake–high precipitation^d^	2519	2947	2519	3409	52.9	61.9	52.9	71.6
	Intake–drought^e^	2044	2391	2044	2766	42.3	49.5	42.3	57.2
	Requirement	1560	2028	1732	2340	27.9	55.1	47.1	37.8
	Balance–high precipitation^d^	959	919	787	1069	25.0	6.8	5.8	33.8
	Balance–drought^e^	484	363	312	426	14.4	-5.6	-4.8	19.4

^a^ Calculated from forage N, moisture content and desert bighorn sheep diet under average precipitation during treatment.

^b^ Water requirements based on Degen [78]; N requirements based on Herbert [79] and DeYoung [80].

^c^ Calculated from forage N, moisture content and desert bighorn sheep diet under drought conditions during treatment.

^d^ Calculated from forage N, moisture content and desert bighorn sheep diet under above-average precipitation during treatment.

^e^ Calculated from forage N and moisture content under drought conditions during pretreatment, and desert bighorn sheep diet during above-average precipitation during treatment.

In the study area, precipitation across the study period exhibited high temporal variation relative to long-term averages. During the study, early summers had average precipitation, whereas late summers experienced drought conditions. Precipitation exhibits extremely low variability during early summer in this region, because the area typically receives very little rainfall (often none) in this season, and thus, the SPI measure of average precipitation, likely represents drought conditions. During the treatment period, precipitation in autumn and winter was two to five times higher than the long-term average, whereas during much of the pretreatment period the area was under drought conditions during these seasons. Therefore, our estimates of water and nutrient balances in autumn and winter during treatment may not be representative of normal forage conditions for DBS. Thus, for these seasons, to test how DBS might respond under drought conditions without the availability of surface water, we substituted seasonal moisture and N content estimates for each species from the pretreatment drought period with seasonal DBS diet proportions measured during the treatment period, thereby representing the most extreme water-stress scenario for DBS. However, we could not account for the possibility that DBS diets without supplemental water during autumn and winter may have been different under drought conditions, when compared to above-average precipitation.

For seasons when a negative daily water balance was accompanied by a positive N balance, we examined if DBS could potentially meet their daily water and nutrient requirements by shifting part of their diet from forage species with lower moisture content to higher moisture species. For early summer, we systematically reduced the proportion in the diet of the lowest moisture species (i.e., big galleta [31%] and brittlebush [36%]) by 3% (i.e., percent of total diet), and shifted this to the three highest moisture species in DBS diets (i.e., barrel cactus [90%], desert agave [78%] and fishhook cactus [66%]), thereby increasing each of these species in the early summer DBS diet by 1%. We continued in increments of 3%, and at each stage adjusted estimates of DBS water and N intake accordingly. Shifting from the lowest moisture species ceased if the percentage of that species in DBS diet decreased to below 1%. The species with the next lowest moisture content was then selected for reduction (i.e., in this case Wright’s buckwheat [37%]). Diet shifts were to the two highest moisture species in late summer (i.e., barrel cactus [93%] and fishhook cactus [66%]) and autumn (i.e., janusia [71%] and fishhook cactus [60%]), because only two higher moisture species occurred in DBS diets in these seasons. Despite having a high moisture content in all seasons, ocotillo was excluded from diet shifts because their leaves are only available for a short period following rains, and stems are not consumed by DBS. In winter, diet shifts were from the species in DBS diets with the lowest N content (i.e., big galleta [0.7%]) to the species with the highest N content (i.e., fairy duster [3.3%]).

**Fig 3:**
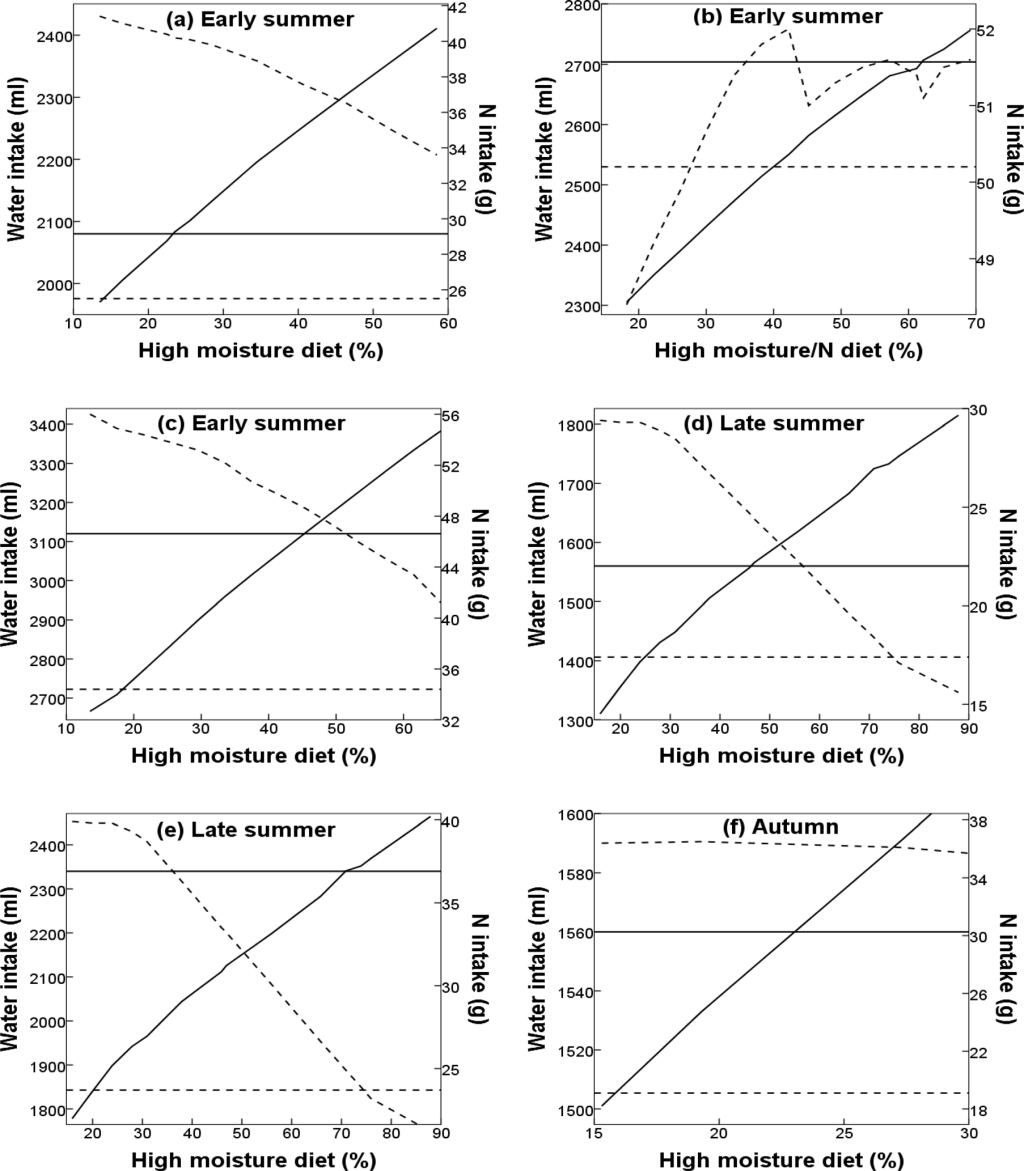
**Seasonal water (ml; solid line) and nitrogen (N; g; dashed line) intake of desert bighorn sheep (DBS) under average precipitation for a) non-reproductive and early breeding females, b) late breeding females, and c) males, and under drought conditions for d) non-reproductive and reproductive females, e) males, f) early breeding females, g) non-reproductive and late breeding females, h) males, i) early breeding females, and j) late breeding females in response to shifts in diet in Cabeza Prieta National Wildlife Refuge, Arizona, USA**. Panels f to j are calculated from forage moisture and N content in pretreatment under drought conditions, and DBS diet in treatment under above-average precipitation. The start of lines at the left represent observed diet proportions (i.e., without shifts). Horizontal lines represent DBS daily maintenance requirements for water (solid) and N (dashed), and thus intakes above these lines represent a positive balance.

The diet of non-reproductive and early breeding female DBS in early summer would have to include 23% higher moisture species for them to be able to attain a positive water balance, while still maintaining a positive N balance; this equates to a shift in their diet of 10% (Fig 3A). Males would need to shift their diet by 33%, to 46% higher moisture species consumed to meet their water and N requirements in early summer (Fig 3C). To overcome the larger water deficit facing DBS in late summer, diets of all females would have to include 47% higher moisture forages (31% diet shift; Fig 3D) and male diets would have to include 71% higher moisture species (55% diet shift; Fig 3E).

We estimated a deficit in daily water and N intake for late breeding female DBS in early summer in the absence of surface water (Table 1). We examined whether a female nursing a lamb during this season could meet her water and N requirements through shifts in forage consumption. In this case, if the female shifted her diet, not only from low to higher moisture forages, but concurrently a portion from low to higher N forages, she could potentially meet her daily water and N requirements with a diet shift of 44% (Fig 3B).

In autumn under drought conditions, we predicted that female and male DBS would be able to meet their daily N requirements, but unable to meet their daily water requirements through forage alone (Table 1). By shifting part of their diets from low to high moisture forage species, we estimated that non-reproductive and late breeding females could achieve a positive water balance in autumn if their diets included 23% higher moisture forages (8% diet shift; Fig 3F). Male and early breeding female DBS diets in autumn would need to include 59% higher moisture species to meet their daily water requirements (44% diet shift; Fig 3G and 3H). During winter under drought conditions, we predicted that reproductive female DBS (i.e., early and late breeders) would be able to obtain enough water from their forage to meet their daily requirements; however, we estimate that they would have a N deficit (Table 1). To overcome this negative balance, early and late breeding female diets would have to include 18% and 7% higher N forages, respectively (early breeders – 17% diet shift, Fig 3I; late breeders – 6% diet shift; Fig 3J).

## Discussion

Comparison between treatment (SP) and control (CP) ranges during the treatment period, indicates that, under the climatic conditions of this study, the removal of supplemental water from DBS range had little effect on DBS diet composition in all seasons. Diet quality also showed little variation between SP and CP in the treatment period (although there were some differences in N and moisture content of DBS diets in some seasons during pretreatment). Forb consumption by DBS increased over the course of the study; however, preference for forbs (i.e., Jacobs D values) generally remained unchanged. This was likely due to increasing availability of forbs as a result of higher than average precipitation later in the study. Grasses are low quality forages, being low in both moisture and protein content. Grass consumption by DBS on both ranges decreased significantly during late summer, likely due to the tendency for grasses to become drier and senesce more rapidly than other forage types; however, grasses comprised a very small portion of DBS diets in all seasons.

The similarity of DBS diets between ranges during the treatment period is contrary to findings from other ungulate studies that reported that the availability of surface water influenced foraging decisions. For example, during the hot season, when springbok had access to drinking water, they primarily consumed grasses, whereas without water available, they achieved their water balance by selecting flowers, seeds, and leaves of shrubs, and did so before dawn, when these forages were most succulent [88]. Captive studies on Nubian ibex (*Capra nubiana*) by Hochman and Kotler [89] also concluded that availability of water plays an important role in ibex foraging decisions. Our results suggest that DBS, at least under the climatic conditions of this study, do not respond to water availability by altering their foraging behavior, and thus, wildlife water developments may not influence DBS diets. However, the precipitation patterns that occurred during this study did not allow us to measure DBS diet selection in the absence of supplemental water provision during drought in autumn and winter. Based on the measurements in this study, we estimated that in the absence of a permanent water source and under drought conditions (i.e., for autumn and winter, applying pretreatment drought forage quality to treatment diet composition under wet conditions), female and male DBS would be unable to meet their daily water requirements from preformed water in their forage and metabolic water production in all seasons, except winter. Furthermore, when precipitation was below average, early breeding females had a N deficit in winter, and late breeding females had a N deficit in winter and early summer. However, available forages during these seasons and under drought conditions contained sufficient moisture and N levels, such that we predicted that by shifting their diets to higher quality forage species (i.e., higher moisture and/or N content), DBS could potentially obtain their daily water and nutrient needs without drinking water.

Other ungulates in arid regions overcome seasonal water shortfalls by altering their diets. Dorcas gazelles (*Gazella dorcas*) in the Negev Desert in Israel augmented their water intake in summer by increasing foraging effort on succulent plants [90]. Arabian oryx, typically bulk grazers that feed primarily on grasses, were observed browsing trees during the dry season [91] and digging extensively for forbs and roots (Tear, pers. obs. in [92]). Other species of bulk feeders in arid environments also readily select browse during the dry seasons [93, 94]. Manser and Brotherton [2] reported that dik-diks selected forage species that they avoided in the wet season to meet their minimum daily water requirements during the dry season, and that forage species preferences in the dry season were best explained by relative abundance and moisture content. Thus, given that there is adequate availability of forages with sufficient moisture levels, and ungulate densities are below carrying capacity, some species have the capacity to survive their most water-stressed seasons through diet selection.

Altering their diet to balance water and nutrients is an important strategy for DBS, given their fragmented habitat. Ungulates that have limited or no access to free-standing water, will often disperse to areas that have received recent precipitation to capitalize on the flush of vegetation [95, 96]. Desert bighorn sheep inhabit isolated mountain ranges, and thus, dispersal is generally not a viable option. Therefore, they must subsist on forages that are available within their range. The diversity and seasonal variability of DBS diets [87, 97, 98] suggest that DBS are well-adapted to making resourceful foraging decisions to cope during water- and nutrient-stressed periods.

Monson and Sumner [39] submit that if moisture content of DBS diets is sufficiently high, they can probably obtain all of their exogenous water preformed in their forage, and thereby become independent of permanent water sources. Indeed, even under drought conditions, succulents in our study area maintained high moisture levels (61–93%) even during the dry summer seasons, indicating that succulents can serve as a superior source of dietary water during the hottest, driest time of year. Warrick and Krausman [99] reported that when surface water is unavailable, DBS will consume barrel cactus to supplement their water intake during the most water-stressed seasons. Watts [27] also found high cacti consumption by DBS (18% of annual diet), which he attributed to an adaptation for increasing preformed water intake during periods of heat stress. Another study reported that DBS consumption of agave increased from < 3% stalks used during average to above-average precipitation years, to 20–53% utilization during dry years [100]. In our study, the overall proportion of succulents in DBS diets across ranges, seasons, and treatments ranged from 10% to 25%. We determined that DBS may be able to overcome seasonal water and nutrient deficits by shifting their diets to include more forages with higher moisture and/or N levels. Our estimates showed high seasonal variation in water and N deficits among non-reproductive females, reproductive females (early and late breeders), and males. During seasons when DBS were in a water deficit, the proportions of high moisture forages in DBS diets required to gain a positive water balance ranged from 23% to 71% (i.e., diets shifts of 8–55%). When examining increased amounts of these species in DBS diets, we have not considered levels of secondary compounds these forages may contain, which could potentially be harmful to DBS. With the exception of janusia, these high moisture species are succulents (i.e., agave, barrel cactus, and fishhook cactus). Succulents can contain high levels of oxalic acid, which can cause renal toxicity in ruminants when ingested in larger quantities [101], and thus, these high proportions of succulents in DBS diets could be toxic. However, the rumen environment of sheep and goats rapidly adapts to increasing levels of oxalic acid with increased capacity to degrade it, thereby preventing poisoning [102, 103]. Indeed, Watts [27] reported a proportion of succulents in DBS diets in excess of 60% during one month. Therefore, DBS may have the physiological capacity to shift their diets and consume high quantities of succulents in an attempt to achieve water balance. However, succulents typically contain low levels of N, and thus, for DBS to include large proportions in their diet, they must already have a sufficient surplus of N in their diet.

Actual water balances of DBS may be higher than what we estimated in this study. In some regions, water content of aridland plants has been shown to increase markedly at night as air cools and relative humidity increases [104, 105]; however, other studies in Arizona [106] and in our study area [4] did not find increased moisture content of DBS forages at night. However, it has been reported that during summer, DBS spend more time feeding at night [106, 107], and this nocturnal activity could decrease water requirements through reduced heat loads. This change in feeding behavior has been demonstrated in springbok, where in summer they shifted foraging to night and early morning [108]. Furthermore, our estimates of DBS water intake may be conservative. An important assumption in this study, is that there was no surface water available to DBS in all seasons, which is unrealistic, even during severe drought years. It is highly likely that DBS on the treatment range had some access to ephemeral sources of drinking water, at least during autumn and winter, and perhaps periodically during late summer in small potholes after rains. Also, when microhistological fecal analyses are used in dietary studies, higher moisture species may be underrepresented because of their high digestibility [109], and therefore, DBS may have been consuming more higher moisture forages than we estimated. Finally, our water and N intake for male DBS may be underestimated, because we used diet composition from females when calculating male intakes, and it has been reported that male DBS have higher quality diets than females [35, 97, 98].

Desert bighorn sheep have evolved to live in the desert, and have many physiological adaptations that allow them to cope with water deprivation. They can respond to water deprivation by decreasing water loss from the rumen through a reduction in rumen volume, decreasing body weight, and thus loss of body water, increasing extraction of water from feces, and concentrating urine [9, 80]. Desert bighorn sheep also have a superior ability to rebound from dehydration by rapidly rehydrating when free-standing water becomes available; they can consume more than 20% of their bodyweight in a single visit to a water source [110]. Therefore, DBS are resilient to periods of water deprivation, and although daily water deficits extrapolate to 5 l to 51 l over a season (which in the upper extent of this range could be lethal), with the exception of early summer, it would be rare for no rainfall to occur over an entire season, and thus, DBS would rarely reach these levels of water deficit. Therefore, when periodic precipitation provides occasional drinking opportunities when potholes and tinajas fill with water, DBS could quickly recover from dehydration, and thus, they may not actually require substantial diet shifts to survive water-stressed periods.

It is essential to synchronize the period of highest resource demand (i.e., for female ungulates, late gestation and lactation) with the period of peak forage abundance and nutritional value [79, 111]. With the extended breeding season of DBS, combined with the unpredictability of precipitation in arid regions, we wanted to examine whether there was any timing of breeding advantage (i.e., early vs late) for a desert bighorn female for achieving water and nutrient balance. When early and late breeding females were compared across seasons where they experienced negative water balances (i.e., early summer to autumn), the mean proportion of higher moisture forages required in their diets to overcome these shortfalls did not differ, suggesting that timing of breeding does not influence a female’s ability to meet her daily water requirements. However, early breeders had a greater nutrient deficit in winter than late breeders, requiring 18% higher N forages compared to 7% for late breeders. Therefore, it may be more advantageous for a desert bighorn female to breed later, such that lambing coincides with a time when forages have more beneficial N levels. Additionally, lambs start foraging more intensively during late lactation, and those born to late-breeding females would be able to capitalize earlier in their development on the significant increase in forage quality that occurs in autumn.

When testing whether hypothetical shifts in DBS diets would allow DBS to survive on water and nutrients obtained from forage alone, we assume DBS densities are sufficiently low such that forage is not limited; most DBS populations in the southwestern United States are small (i.e., < 100 individuals; [112]). We consider only the primary elements necessary in a DBS diet; water and protein. We have not considered secondary elements required by DBS (e.g., metals, salts, electrolytes, etc.); we assume these elements will be found in DBS diets in suitable levels or can be obtained outside of diet (e.g., licks). We also do not account for energetic costs of searching for, acquiring, and handling alternate forages. In this study, we only examine whether DBS can potentially meet water and N requirements without surface water, and offer no opinions regarding how the absence of free water may influence other demographic rates (e.g., fecundity).

Unpredictable precipitation patterns present challenges to ungulates inhabiting arid climates. Desert ungulates cope with their xeric and often nutrient-stressed environment through physiological adaptations and behavioral modifications. Given the availability of quality forage (i.e., suitable moisture and protein content), ungulates on arid lands can make foraging decisions that allow them to survive during the hottest and driest seasons. The DBS serves as an excellent example of how an ungulate can adapt to survive in an arid environment. This study revealed that, based on observed diets, DBS would not be able to meet their daily water requirements in some seasons and under some climatic conditions in the absence of surface water. However, we have demonstrated that resourceful foraging decisions could potentially allow DBS to overcome seasonal nutrient deficits, and concurrently gain a positive water balance solely from the forage they consume. Our findings raise questions about the efficacy, or in fact necessity, of the management practice of providing supplemental water to DBS during water-stressed periods (at least under the climatic conditions observed during our study). Indeed, projected temperature increases and decreasing precipitation over the coming century will undoubtedly alter DBS water requirements and forage moisture and nutrient content, and thus, could further compromise DBS ability to subsist solely on forage. This study only addressed water and nutrient requirements for adult survival, and it is unknown whether these conditions can facilitate population growth. Therefore, future research should investigate how water provision may also affect other demographic rates that ultimately lead to population growth.

## References

[1] Van Soest PJ Nutritional ecology of the ruminant 6th ed. Ithaca: Cornell University Press 1994.

[2] Manser MB, Brotherton PNM. Environmental constraints on the foraging behaviour of a dwarf antelope (*Madoqua kirkii*). Oecologia. 1995; 102: 404–412. 10.1007/BF00341352 28306883

[3] Everitt JH, Gonzalez CL. Seasonal nutrient content of food plants of white-tailed deer on the south Texas plains. J Range Manage. 1981; 34: 506–510.

[4] Fox LM. Nutritional content of forage in Sonoran pronghorn habitat, Arizona. M.Sc. Thesis, University of Arizona. 1997.

[5] Owen-Smith N, Mason DR, Ogutu JO. Correlates of survival rates for 10 African ungulate populations: density, rainfall and predation. J Anim Ecol. 2005; 74: 774–788.

[6] Marshal JP, Cain JW, Bleich, VC, Rosenstock SS. Intrinsic and extrinsic sources of variation in the dynamics of large herbivore populations. Can J Zool. 2009; 87: 103–111.

[7] Augustine DJ. Response of native ungulates to drought in semi-arid Kenyan rangeland. Afr J Ecol. 2010; 48: 1009–1020.

[8] Ismail K, Kamal K, Plath M, Wronski T. Effects of an exceptional drought on daily activity patterns, reproductive behaviour, and reproductive success of reintroduced Arabian oryx (*Oryx leucoryx*). J Arid Environ. 2011; 75: 125–131.

[9] Cain J III, Krausman PR, Rosenstock SS, Turner JC. Mechanisms of thermoregulation and water balance in desert ungulates. Wildl Soc Bull. 2006; 34: 570–581.

[10] Degen AA, Kam M, Hazan A, Nagy KA. Energy expenditure and water flux in three sympatric desert rodents. J Anim Ecol. 1986; 55: 421–429.

[11] Ostrowski S, Williams JB, Bedin E, Ismail K. Water influx and food consumption of free-living oryxes (*Oryx leucoryx*) in the Arabian Desert summer. J Mamm. 2002; 83: 665–673.

[12] Taylor CR. The minimum water requirements of some East African bovids. Symp Zool Soc London. 1968; 21: 195–206.

[13] Hofmeyr MD, Louw GN. Thermoregulation, pelage conductance and renal function in the desert-adapted springbok, *Antidorcas marsupialis*. J Arid Environ. 1987; 13: 137–151.

[14] Taylor CR. The eland and oryx. Sci Amer. 1969; 220: 88–95. 5761730 10.1038/scientificamerican0169-88

[15] Hoppe PP. How to survive heat and aridity: ecophysiology of the dik-dik antelope. Vet Med Rev. 1977; 8: 77–86.

[16] Lewis JG. Game domestication for animal production in Kenya: activity patterns of eland, oryx, buffalo and zebu cattle. J Agric Sci Cambridge. 1977; 89: 551–563.

[17] Knight MH, Knight-Eloff AK, Bornman JJ. The importance of borehole water and lick sites to Kalahari ungulates. J Arid Environ. 1988; 15: 269–281.

[18] Broyles B. Desert wildlife water developments: questioning use in the southwest. Wildl Soc Bull. 1995; 23: 663–675.

[19] Rosenstock SS, Ballard W, deVos J. Benefits and impacts of wildlife water developments. J Range Manage. 1999; 52: 302–311.

[20] Krausman PR, Rosenstock SS, Cain JW III. Developed waters for wildlife: science, perception, values, and controversy. Wildl Soc Bull. 2006; 34: 563–569.

[21] Epaphras AM, Gereta E, Lejora IA, Ole Meing’ataki GE, Ng’umbi G, Kiwango Y, et al Wildlife water utilization and importance of artificial waterholes during dry season at Ruaha National Park, Tanzania. Wetlands Ecol Manage. 2008; 16: 183–188.

[22] Ayeni JSO. Utilization of waterholes in Tsavo National Park (East). E Afr Wildl. 1975; 13: 305–323.

[23] Kalikawa MC. Baseline vegetation description at artificial watering points of Central Kalahari Game Reserve. Afr J Ecol. 1990; 28: 253–256.

[24] Monson G. Water requirements. Desert Bighorn Counc Trans. 1958; 2: 64–66.

[25] Weaver RA, Hall JM. Desert bighorn sheep in southeastern San Bernadino County. CA Dept Fish Game. 1971.

[26] Mendoza J. Status of the desert bighorn in Sonora. Desert Bighorn Counc Trans. 1976; 20: 25–26.

[27] Watts TJ. Detrimental movement patterns in a remnant population of bighorn sheep (*Ovis canadensis mexicana*). M.S. Thesis, New Mexico State University. 1979.

[28] Krausman PR, Torres S, Ordway LL, Hervert JJ, Brown M. Diel activity of ewes in the Little Harquahala Mountains, Arizona. Desert Bighorn Counc Trans. 1985; 29: 24–26.

[29] McQuivey RP. The desert bighorn sheep in Nevada. NV Dept Wildl Biol Bull. 1978; 6: 1–81.

[30] Leslie D, Douglas C. Desert bighorn sheep of the River Mountains, Nevada. Wildl Monogr. 1979; 66.

[31] Olech LA. Summer activity rhythms of peninsular bighorn sheep in Anza-Borrego Desert State Park, San Diego County, California. Desert Bighorn Counc Trans. 1979; 23: 33–36.

[32] Cunningham SC. Aspects of the ecology of Peninsular desert bighorn sheep (*Ovis canadensis cremnobates*) in Carrizo Canyon, California. M.Sc. Thesis, Arizona State University. 1982.

[33] Bristow KD, Wennerlund JA, Schweinsberg RE, Olding RJ, Lee RE. Habitat use and movements of desert bighorn sheep near the Silver Bell Mine, Arizona. AZ Game Fish Dept Res Tech Rep. 1996; 25.

[34] Turner JC, Douglas CL, Hallum CR, Krausman PR, Ramey RR. Determination of critical habitat for the endangered Nelson’s bighorn sheep in southern California. Wildl Soc Bull. 2004; 32: 427–448.

[35] Bleich VC, Boyer RT, Wehausen JD. Sexual segregation in mountain sheep: resources or predation? Wildl Monogr. 1997; 134: 1–50.

[36] Andrews NG, Bleich VC, August PV. Habitat selection by mountain sheep in the Sonoran Desert: implications for conservation in the United States and Mexico. CA Wildl Conserv Bull. 1999; 12: 1–17.

[37] Oehler MW Sr, Bleich VC, Bowyer RT, Nickolson MC. Mountain sheep and mining: implications for conservation and management. CA Fish Game. 2005; 91: 149–178.

[38] Sappington JM, Longshore KM, Thompson, DB. Quantifying landscape ruggedness for animal habitat analyses: a case study using desert bighorn in the Mojave desert. J Wildl Manage. 2007; 71: 1419–1426.

[39] Monson G, Sumner L. The desert bighorn: its life history, ecology, and management Tucson: University of Arizona Press 1990.

[40] Valdez R, Krausman PR. Description, distribution, and abundance of mountain sheep in North America In: Valdez R, Krausman PR editors. Mountain sheep of North America. Tucson: University of Arizona Press 1999 pp 3–22.

[41] Hansen CG. Habitat In: Monson G, Sumner L, editors. The desert bighorn: it’s life history, ecology, and management Tucson: University of Arizona Press 1990 pp 64–79.

[42] Krausman PR, Sandoval A, Etchberger RC. Desert bighorn sheep: natural history In: Valdez R, Krausman PR, editors. Mountain sheep of North America. Tucson: University of Arizona Press 1999 Pp 139–191.

[43] McKinney T, Smith TW. Diets of adults and lambs of desert bighorn sheep during years of varying rainfall in central Arizona. Southwest Nat. 2007; 52: 520–527.

[44] Wishart W. Bighorn Sheep In: Schmidt JL, Gilbert DL, editors. Big game of North America: ecology and management. Harrisburg: Stackpole Books 1978 pp 161–171.

[45] Shackleton DM. Ovis canadensis. Mamm Sp. 1985; 230: 1–9.

[46] Krausman PR, Hervert JJ, Ordway LL. Capturing deer and mountain sheep with a net gun. Wildl Soc Bull. 1985; 13: 71–73.

[47] Sikes RS, Gannon WL, Animal Care and Use Committee of the American Society of Mammalogists. Guidelines of the American Society of Mammalogists for the use of wild mammals in research. J Mamm. 2011; 92: 235–253. 10.1093/jmammal/gyw078 PMC5909806 29692469

[48] Western Regional Climate Data Center. 2005. Arizona climate summaries. Available: http://www.wrcc.dri.edu/cgi-bin/cliMAIN.pl?aztacn. Accessed 15 February 2006.

[49] McKee TB, Doesken NJ, Kilest J. The relationship of drought frequency and duration to time scales. Proc 8^th^ Conf Appl Climatol. 1993. pp 179–186.

[50] Guttman NB. Accepting the standardized precipitation index: a calculation algorithm. J Amer Water Resource Assoc. 1999; 35: 311–322.

[51] National Drought Mitigation Center. University of Nebraska–Lincoln, Lincoln, Nebraska, USA. 2014. Available: http://drought.unl.edu/MonitoringTools/DownloadableSPIProgram.aspx.

[52] Cain JW III, Krausman PR, Morgart JR, Jansen BD, Pepper MP. Responses of desert bighorn sheep to removal of water sources. Wildl Monogr. 2008; 171.

[53] Stewart-Oaten AJ, Bence JR, Osenbert CW. Assessing effects of unreplicated perturbations: no simple solutions. Ecology. 1992; 73: 1396–1404.

[54] Stewart-Oaten AJ, Murdoch WW, Parker KR. Environmental impact assessment: “pseudoreplication” in time? Ecology. 1986; 67: 929–940.

[55] Canfield RH. Application of the line intercept method in sampling range vegetation. J Forestry. 1941; 38: 388–394.

[56] Etchberger RC, Krausman PR. Evaluation of five methods for measuring desert vegetation. Wildl Soc Bull. 1997; 25: 604–609.

[57] Fracker SB, Brischle HA. Measuring the local distribution of ribes. Ecology. 1944; 25: 283–303.

[58] Sparks DR, Malechek JC. Estimating percentage dry weight in diets using a microscopic technique. J Range Manage. 1968; 21: 264–265.

[59] Holechek JL, Vavra M. The effects of slide and frequency observation numbers on the precision of microhistological analysis. J Range Manage. 1981; 34: 337–338.

[60] Krausman PR, Leopold BD, Seegmiller RF, Torres SG. Relationships between desert bighorn sheep and habitat in western Arizona. Wildl Monogr. 1989; 102.

[61] Fitzgerald AE, Waddington DC. Comparison of two methods of fecal analysis of herbivore diet. J Wildl Manage. 1979; 43: 468–473.

[62] Holechek JL, Vavra M, Pieper RD. Botanical composition determination of range herbivore diets: a review. J Range Manage. 1982; 35: 309–315.

[63] Gill RB, Carpenter LH, Bartmann RM, Baker DL, Schoonveld GG. Fecal analysis to estimate mule deer diets. J Wildl Manage. 1983; 47: 902–915.

[64] Jacobs J. Quantitative measurement of food selection: A modification of the forage ratio and Ivlev’s electivity index. Oecologia. 1974; 14: 413–417. 10.1007/BF00384581 28308662

[65] Ivlev VS. Experimental ecology of the feeding of fishes New Haven: Yale University Press 1961.

[66] Lechowicz MJ. The sampling characteristics of electivity indices. Oecologia. 1982; 52: 22–30. 10.1007/BF00349007 28310104

[67] Rautenstrauch KR, Krausman PR, Whiting FM, Brown WH. Nutritional quality of desert mule deer forage in King Valley, Arizona. Desert Plants. 1988; 8: 172–174.

[68] Krausman PR, Ordway LL, Whiting FM, Brown WH. Nutritional composition of desert mule deer forage in the Picacho Mountains, Arizona. Desert Plants. 1990; 10: 32–34.

[69] Seegmiller RF, Krausman PR, Brown WH, Whiting FM. Nutritional composition of desert bighorn sheep forage in the Harquahala Mountains, Arizona. Desert Plants. 1990; 10: 87–90.

[70] Bleich VC, Bowyer RT, Clark DJ, Clark TO. An analysis of forage used by mountain sheep in the eastern Mojave Desert, California. Desert Bighorn Counc Trans. 1992; 36: 41–47.

[71] Marshal JP, Krausman PR, Bleich VC. Rainfall, temperature, and forage dynamics affect nutritional quality of desert mule deer forage. Rangeland Ecol Manage. 2005; 58: 360–365.

[72] Martinez-Aguilar JF, Pena-Alvarez A. Characterization of five typical agave plants used to produce mezcal through their simple lipid composition analysis by gas chromatography. J Agric Food Chem. 2009; 57: 1933–1939. doi: 10.1021/jf802141d 19216532 10.1021/jf802141d

[73] Karsch RC. Desert bighorn sheep adult female and lamb survival, cause-specific mortality, and parturient female habitat selection in the Peloncillo Mountains, New Mexico, USA. M.Sc. Thesis, New Mexico State University. 2014.

[74] Russo JP. The desert bighorn in Arizona. AZ Game Fish Dept Bull. 1956; 1.

[75] Witham JH. Desert bighorn sheep in southwestern Arizona. Ph.D. Thesis, Colorado State University. 1983.

[76] Mazaika R, Krausman PR, Etchberger RC. Forage availability for mountain sheep in Pusch Ridge Wilderness, Arizona. Southwest Nat. 1992; 37: 372–378.

[77] Weston RH. Factors limiting the intake of feed by sheep. XIII Voluntary roughage consumption in late pregnancy and early lactation in relation to protein nutrition. Aust J Agric Res. 1988; 39: 679–689.

[78] Houpt TR. Water, electrolytes, and acid-base balance In: Swenson MJ, editor. Dukes’ physiology of domestic animals, 8th ed. Ithaca: Cornell University Press 1970 pp 743–766.

[79] Robbins CT. Wildlife feeding and nutrition, 2nd ed. San Diego: Academic Press Inc 1993.

[80] Turner JC. Water, energy and electrolyte balance in the desert bighorn sheep, *Ovis canadensis*. Ph.D. Thesis, University of California–Riverside. 1973.

[81] Degen AA. Fat-tailed Awassi and German mutton merino sheep under semi-arid conditions. 2. Total body water and water turnover during pregnancy and lactation. J Agric Sci Cambridge. 1977; 88: 699–704.

[82] Hebert DM. Altitudinal migration as a factor in the nutrition of bighorn sheep. Ph.D. Thesis, University of British Columbia. 1973.

[83] DeYoung RW, Hellgren EC, Fulbright TE, Robbins WF Jr, Humphreys ID. Modeling nutritional carrying capacity for translocated desert bighorn sheep in western Texas. Restor Ecol. 2000; 8: 57–65.

[84] Zar JH. Biostatistical analysis, 3rd ed. Upper Saddle River: Prentice Hall 1996.

[85] IBM Corp. 2012 IBM SPSS statistics for Windows, version 21.0. Armonk: IBM Corp.

[86] Fulbright TE, Robbins WF, Hellgren EC, DeYoung RW, Humphreys ID. Lack of diet partitioning by sex in reintroduced desert bighorn sheep. J Arid Environ. 2001; 48: 49–57.

[87] Tarango LA, Krausman PR, Valdez R, Katting RM. Research observation: desert bighorn sheep diets in northwestern Sonora, Mexico. J Range Manage. 2002; 55: 530–534.

[88] Nagy KA, Knight MH. Energy, water, and food use by springbok antelope (*Antidorcas marsupialis*) in the Kalahari Desert. J Mamm. 1994; 75: 860–872.

[89] Hochman V, Kotler BP. Effects of food quality, diet preference and water on patch use by Nubian ibex. Oikos. 2006; 112: 547–554.

[90] Baharav D, Rosenzweig ML. Optimal foraging in Dorcas gazelles. J Arid Environ. 1985; 9: 167–171.

[91] Tear TH. Range use patterns and the development of a natural grazing system in reintroduced Arabian oryx (*Oryx leucoryx*) in the Sultanate of Oman. M.S. Thesis, University of Idaho. 1992.

[92] Tear TH, Mosley JC, Ables ED. Landscape-scale foraging decisions by reintroduced Arabian oryx. J Wildl Manage. 1997; 61: 1142–1154.

[93] Mills MGL, Retief PF. The response of ungulates to rainfall along the riverbeds of the southern Kalahari 1972–1982. Koedoe Suppl. 1984 pp 129–141.

[94] Owen-Smith N. Foraging responses of kudus to seasonal changes in food resources: elasticity in constraints. Ecology. 1994; 75: 1050–1062.

[95] Corp N, Spalton A, Gorman ML. The influence of rainfall on range in a female desert ungulate: the Arabian oryx (*Oryx leucoryx*) in the Sultanate of Oman. J Zool. 1998; 246: 369–377.

[96] Attum O, Ghazali U, El NOby SK, Hassan IN. The effects of precipitation history on the kilometric index of Dorcas gazelles. J Arid Environ. 2014; 102: 113–116.

[97] Miller GD, Gaud WS. Composition and variability of desert bighorn sheep diets. J Wildl Manage. 1989; 53: 597–606.

[98] Brewer CE, Harveson L. Diets of bighorn sheep in the Chihuahuan Desert, Texas. Southwest Nat. 2007; 52: 97–103.

[99] Warrick GD, Krausman PR. Barrel cacti consumption by desert bighorn sheep. Southwest Nat. 1989; 34: 483–486.

[100] Harkleroad D, Krausman PR. Use of agave by desert bighorn sheep. Southwest Nat. 2014; 59: 272–274.

[101] James LF. 1972. Oxalate toxicosis. Clin Toxicol. 1972; 5: 231–243. 4559019 10.3109/15563657208991002

[102] Duncan AJ, Frutos P, Young SA. Rates of oxalic acid degradation in the rumen of sheep and goats in response to different levels of oxalic acid administration. Anim Sci. 1997; 65: 451–455.

[103] Belenguer A, Ben Bati M, Hervás G, Toral PG, Yáñez-Ruiz DR, Frutos P. Impact of oxalic acid on rumen function and bacterial community in sheep. Animal. 2013; 7: 940–947. doi: 10.1017/S1751731112002455 23298534 10.1017/S1751731112002455

[104] Taylor CR. The minimum water requirements of some East African bovids.–Symp. Zool. Soc. London. 1968 21: 195–206.

[105] Louw GN, Seely MK. Longman: Ecology of desert organisms. 1982.

[106] Alderman JA, Krausman PR, Leopold BD. 1989. Diel activity of female desert bighorn sheep in western Arizona. J Wildl Manage. 1989 53:264–271.

[107] Monson G. Long-distance and nighttime movements of desert bighorn sheep. Desert Bighorn Counc Trans. 1964; 8: 11–17.

[108] Bigalke RC. Observations on the behaviour and feeding habits of the springbok, *Antidorcas marsupialis*. Zool Africana. 1972; 7: 333–359.

[109] Krausman PR, Kuenzi AJ, Etchberger RC, Rautenstauch KR, Ordway LL, Hervert JJ. Diets of desert mule deer. J Range Manage. 1997; 50: 513–522.

[110] Turner JC, Weaver RC. Water In: Monson G, Sumner L, editors. The desert bighorn: its life history, ecology, and management. Tucson: University of Arizona Press 1990 pp 100–112.

[111] Clutton-Brock TH. The evolution of parental care Princeton: Princeton University Press 1991.

[112] Epps CW, McCullough DR, Wehausen JD, Bleich VC, Rechel JL. Effects of climate change on population persistence of desert-dwelling mountain sheep in California. Conserv Biol. 2004; 18: 102–113.

