# Correction: Potential Foraging Decisions by a Desert Ungulate to Balance Water and Nutrient Intake in a Water-Stressed Environment

**DOI:** 10.1371/journal.pone.0154455

**Published:** 2016-04-21

**Authors:** The PLOS ONE Staff

Fig 3 is erroneously truncated. The publisher apologizes for the error. Please see the complete, correct Fig 3 here.

**Fig 3:**
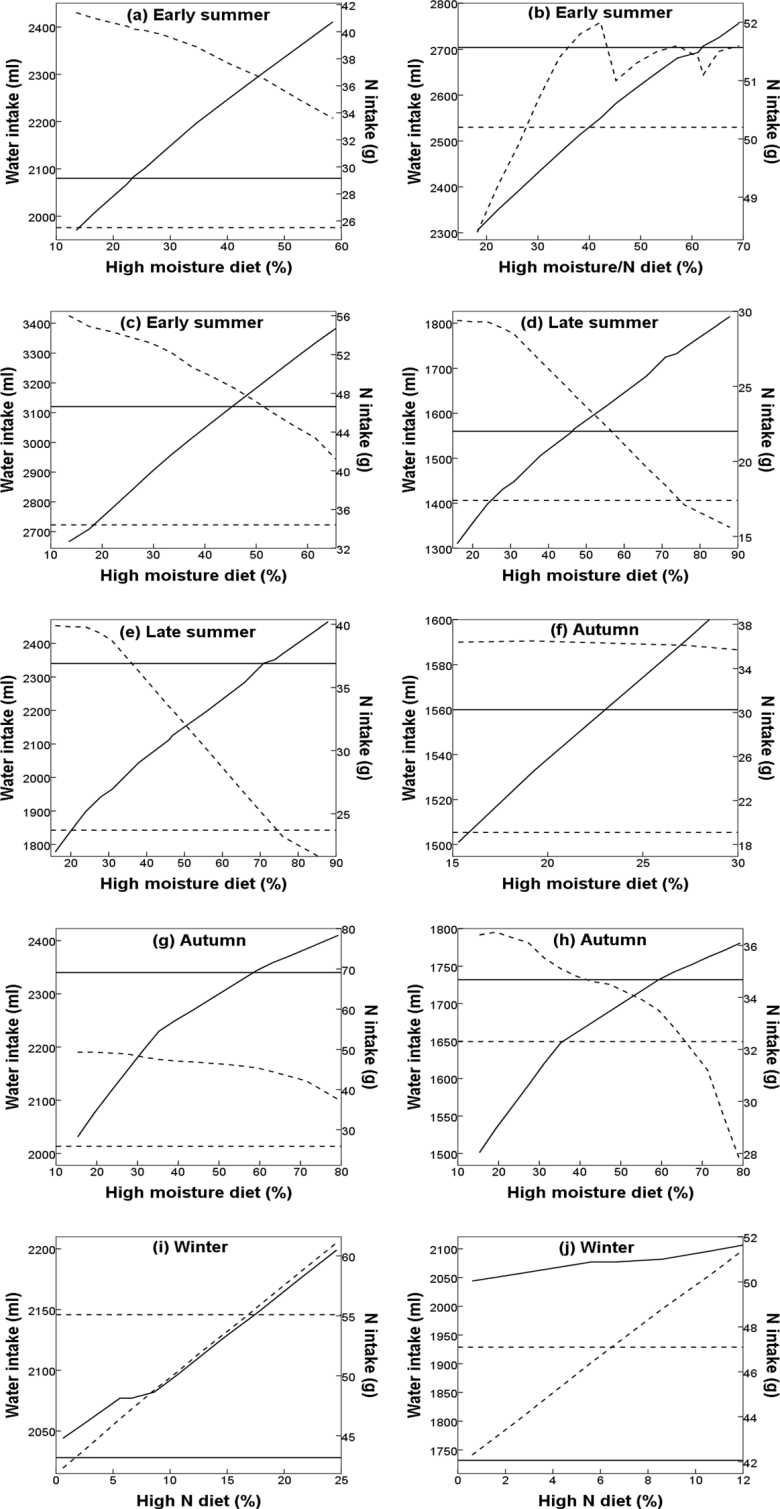
**Seasonal water (ml; solid line) and nitrogen (N; g; dashed line) intake of desert bighorn sheep (DBS) under average precipitation for a) non-reproductive and early breeding females, b) late breeding females, and c) males, and under drought conditions for d) non-reproductive and reproductive females, e) males, f) early breeding females, g) non-reproductive and late breeding females, h) males, i) early breeding females, and j) late breeding females in response to shifts in diet in Cabeza Prieta National Wildlife Refuge, Arizona, USA.** Panels f to j are calculated from forage moisture and N content in pretreatment under drought conditions, and DBS diet in treatment under above-average precipitation. The start of lines at the left represent observed diet proportions (i.e., without shifts). Horizontal lines represent DBS daily maintenance requirements for water (solid) and N (dashed), and thus intakes above these lines represent a positive balance.
